# The effects of high-sensitivity C-reactive protein on the clinical outcomes in obstructive sleep apnea patients undergoing off-pump coronary artery bypass grafting

**DOI:** 10.1186/s12872-021-02168-2

**Published:** 2021-07-31

**Authors:** Mingxin Gao, Kangjun Fan, Wenyuan Yu, Hongli Liu, Yongxiang Wei, Yang Yu

**Affiliations:** 1 Department of Cardiac Surgery, Beijing Anzhen Hospital, Capital Medical University, Beijing, 100029 China; 2 Department of Otolaryngology, Beijing Anzhen Hospital, Capital Medical University, Beijing, 100029 China

**Keywords:** Obstructive sleep apnea, High-sensitivity C-reactive protein, Clinical outcomes, Off-pump cardiac artery bypass grafting

## Abstract

**Purpose:**

To investigate the relationship between obstructive sleep apnea (OSA) severity and high-sensitivity C-reactive protein (Hs-CRP), and their respective impact on the clinical outcomes in patients undergoing off-pump cardiac artery bypass grafting (OPCABG).

**Methods:**

We enrolled consecutive eligible patients listed for elective OPCABG who underwent cardiorespiratory polygraphy before surgery between January 2019 and December 2019 in this prospective observational single-center study. Baseline, intraoperative, and postoperative clinical data were compared between absent-mild and moderate-severe OSA groups. Regression analysis investigated the relationship between Hs-CRP level and severity of OSA, and further assessed the factors influencing postoperative atrial fibrillation, duration of hospitalization, and hospital cost.

**Results:**

Patients with moderate-severe OSA accounted for 42.3% (52/123) of the cohort. Partial pressure of carbon dioxide (PCO_2_), Hs-CRP, apnea hypopnea index (AHI), mean apnea time, maximum apnea time, and oxygen desaturation index ODI ≥ 3% were significantly higher in the moderate-severe OSA group than in the absent-mild OSA group. Left ventricle ejection fraction (LVEF), lowest arterial oxygen saturation (SaO_2_), and mean SaO_2_ were significantly lower in the moderate-severe OSA group. Moderate-severe OSA was associated with elevated Hs-CRP level (OR = 2.356, 95% CI 1.101–5.041, *P* = 0.027). Hs-CRP was an independent risk factor for post-CABG atrial fibrillation (POAF) (OR = 1.212, *P* = 0.01). Hs-CRP level independently correlated with duration of hospitalization (B = 0.456, *P* = 0.001) and hospital cost (B = 1.111, *P* = 0.044).

**Conclusion:**

Hs-CRP level was closely related to OSA severity and have potential utility in predicting POAF, duration of hospitalization, and hospital costs in patients undergoing OPCABG.

## Introduction

Obstructive sleep apnea (OSA) has an incidence of 9–38% in adults and is characterized by repeated or partial obstruction of the respiratory tract during sleep [1]. Intermittent hypoxia induced by OSA could trigger oxidative stress and damage to the vascular endothelium, therefore, it is an independent risk factor for coronary heart disease (CHD) and affects its prognosis [2]. Currently, off-pump cardiac artery bypass grafting (OPCABG) has become one of the primary treatments of CHD [3]. However, there were few reports on the relationship between OSA and post-OPCABG complications, in addition, traditional OSA severity markers, such as apnea hypopnea index (AHI), has limitations in predicting the prognosis of CABG [4].

High-sensitivity C-reactive protein (Hs-CRP) is a classic marker of inflammatory response, which has been proven to be central to the pathogenesis of vascular diseases in the context of OSA. A slight increase in Hs-CRP level could indicate coronary plaque inflammation or coronary artery wall injury, which is closely related to CHD and its associated negative events [5].

In this study, we aimed to investigate the relationship between the severity of OSA and Hs-CRP, and their respective impact on the clinical outcomes in patients undergoing OPCABG during hospitalization.

## Methods

### Study design

This prospective observational single-center study was conducted at the Beijing Anzhen Hospital, Capital Medical University. We enrolled consecutive patients with coronary artery disease who were scheduled to undergo elective OPCABG and had not been diagnosed with OSA from January 2019 to December 2019. The study protocol, which was approved by the institutional review board (Approval No.: 2013025), was explained to all patients, all of whom signed informed consent for sleep monitoring tests (polygraphy, PG).

### Patients

All participating patients, aged between 40 and 75 years old, underwent an overnight sleep study before OPCABG. We excluded patients with valvular disease combined with other heart diseases, central sleep apnea, severe respiratory diseases (e.g., chronic obstructive pulmonary disease), severe diseases of other organs (e.g., renal failure), body temperature > 37.5℃, and preoperative use of morphine and its analogs, sedative drugs, and/or theophylline. We recorded baseline clinical data, including age, sex, body mass index (BMI), body temperature, pre-existing medical conditions (hypertension, diabetes, stroke), history of smoking, blood biochemistry findings, and left ventricular ejection fraction (LVEF) bases on echocardiography, and PG test data.

### PG tests and diagnostic criteria for OSA

Eligible patients were enrolled and admitted to the hospital. PG was performed before OPCABG. Each patient in the sleep monitoring center at Beijing Anzhen Hospital wore a portable sleep monitor (ApneaLink, ResMed, Australia). We used type III PG to detect airflow by nasal catheter, respiratory movement by chest belt, heart rate by electrocardiograph, and arterial oxygen saturation (SaO_2_) by pulse oximetry. All PG test data were analyzed by two physicians at the Sleep Center of Beijing Anzhen Hospital. In case of disagreement between the two, a third physician participated in the data analysis. Sleep apnea was defined as the cessation of airflow through the nose and mouth for > 10 s during sleep; hypopnea, a reduction of > 50% in the airflow intensity and ≥ 4% in SaO_2_ level during sleep. AHI was defined as the total number of apnea or hypopnea episodes per hour during sleep (i.e., AHI = total number of apnea or hypopnea episodes/total sleep duration (min) × 60). Moderate-severe OSA was defined as an AHI ≥ 15/h during a 7-h sleep. Oxygen desaturation index (ODI) ≥ 3% is the number of times that oxygen saturation decreases by > 3% per hour [6]. All moderate-severe patients were recommended for continuous positive airway pressure (CPAP) after discharge.

### OPCABG

All patients underwent OPCABG after the PG test. The same cardiac surgeon performed all surgeries. The number of grafts and surgical duration were recorded. The quality of graft anastomosis met the criteria recommended by the Operation Quality Committee of Beijing Anzhen Hospital. Two ultrasound specialists performed echocardiography in all patients, and a single nurse measured the blood pressure and collected blood samples from the patients. After surgery, the patients were monitored in the intensive care unit (ICU) until ventilator removal was feasible and vital signs were stable; they were discharged from the hospital once they could move freely. The physician in the ICU and the cardiac surgeon determined the ICU stay and hospitalization duration. We recorded the following postoperative data during hospitalization: incidence of major adverse cardiac and cerebrovascular event (MACCE), lung infection, post-CABG atrial fibrillation (POAF); duration of postoperative tracheal intubation; ICU stay; duration of hospitalization, and hospital cost. POAF was defined as the occurrence of AF within 72 h after surgery. Pulmonary infection was defined as a postoperative increase in white blood cell count, obvious inflammation based on postoperative chest radiography and computed tomography, and meeting one of the following conditions: sputum examination reveals new characteristic changes and pathogenic bacterium could be cultivated from blood or respiratory secretions.

### Blinding

The cardiac surgeon, other participating investigators, and research staff were blinded to the findings of the PG tests. After the final enrolled patient was discharged from the hospital in January 2020, all data were revealed to the participating investigators.

### Statistical analysis

All statistical analyses were performed using SPSS, version 24.0 (SPSS Inc., Chicago, IL, USA). Continuous variables with a normal distribution were presented as means ± standard deviations and were compared using independent samples *t*-tests. Continuous variables without a normal distribution were presented as medians (interquartile ranges) and were compared using rank-sum tests. Categorical variables are presented as percentages and were compared using χ^2^ tests. If the missing quantity of measurement data was < 5%, the average value was used to replace the missing value. No missing values in the counting data were noted. Logistic regression analysis was implemented to investigate the relationship between Hs-CRP level (dependent variable) and severity of OSA. To analyze the association between preoperative relevant indicators and POAF, duration of hospitalization and hospital cost, multivariate logistic (dependent variable: POAF) and multiple linear regression analysis (dependent variable: duration of hospitalization and hospital cost) were implemented (sex, age, BMI, hypertension, diabetes, AHI and variables with *P* < 0.1 in the univariate logistic or simple linear regression were included in the model). Two-sided *P* values < 0.05 were considered statistically significant.

## Results

### Baseline data line characteristics

**Fig. 1:**
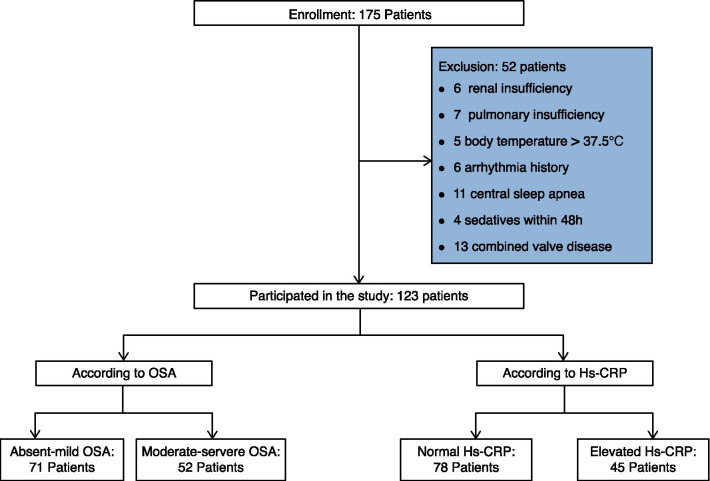
Flow chart showing the inclusion of patients in this study

The study flow chart is shown in Fig. 1. One-hundred-and-seventy-five patients underwent PG and OPCABG from January 2019 to December 2019. A total of 123 patients were included in the final analysis. Based on the PG findings, 71 patients had an AHI < 15 and were assigned to the absent-mild OSA group, while the remaining 52 had an AHI ≥ 15 and were assigned to the moderate-severe OSA group. We set the threshold Hs-CRP level at 2 mg/L, which is according to the results of previous large clinical trials [5]. Seventy-eight patients had an Hs-CRP level of < 2 mg/L and were assigned to the normal Hs-CRP group; 45 had an Hs-CRP level of ≥ 2 mg/L and were assigned to the elevated Hs-CRP group.

**Fig. 2:**
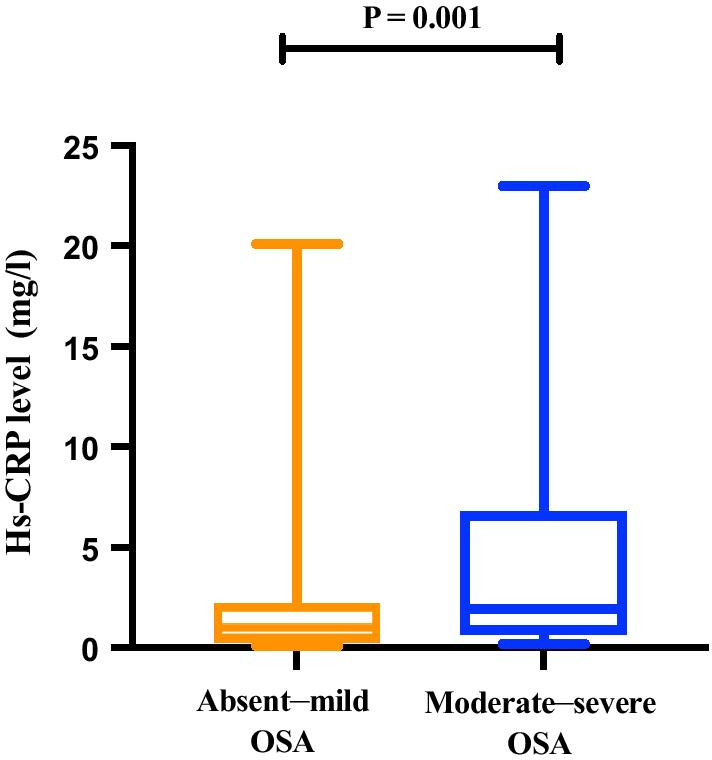
The Hs-CRP level in the moderate-severe OSA group was significantly higher than that in the no-mild OSA group

Preoperative clinical data of the absent-mild OSA and moderate-severe OSA groups were compared (Table 1): partial pressure of carbon dioxide (PCO_2_, 37.1 ± 4.4 vs. 35.5 ± 3.2 mmHg, *P* = 0.033), Hs-CRP (2.0 [0.7, 6.8] vs. 1.1 [0.3, 2.2] mg/L, *P* = 0.001; Fig. 2), AHI (28.5 ± 11.5 vs. 7.1 ± 4.1 events/h, *P* < 0.001), mean apnea time (24 [19, 30] vs. 17 [14, 24] s, *P* < 0.001), maximum apnea time (40 [31, 61] vs. 27 [19, 35] s, *P* < 0.001), and ODI ≥ 3% (23.3 [18.4, 31.1] vs. 6.1 [3.0, 9.3] s, *P* < 0.001), were significantly higher in the moderate-severe OSA group than in the absent-mild OSA group. LVEF (55.9 ± 9.8 vs. 59.5 ± 8.5%, *P* < 0.001), lowest SaO_2_ (81.6 ± 5.8 vs. 87.0 ± 3.7%, *P* < 0.001) and mean SaO_2_ (93.0 ± 2.4 vs. 95.4 ± 2.1%, *P* < 0.001) were significantly lower in the moderate-severe OSA group than in the absent-mild OSA group. No significant difference in other preoperative indexes was found. In addition, the number of grafting performed, duration of surgery, MACCEs, lung infection, POAF, duration of ventilator use, ICU stay, duration of hospitalization, and hospital cost were not significantly different between the two OSA groups (Table 2).

**Table 1:** Baseline clinical data for patients with obstructive sleep apnea (OSA) undergoing off-pump coronary artery bypass grafting (OPCABG)

	Absent-mild OSA (n = 71)	Moderate-severe OSA (n = 52)	*P* value	Total (n = 123)
Sex (male/female)	55/16	39/13	0.750	94/29
Age (years)	61.3 ± 9.3	62.9 ± 8.2	0.334	62.0 ± 8.88
BMI (kg/m^2^)	25.5 ± 3.2	25.2 ± 3.7	0.602	25.4 ± 3.4
Body temperature (˚C)	36.4 ± 0.4	36.3 ± 0.4	0.236	36.3 ± 0.4
Hypertension, n (%)	49 (69)	40 (77)	0.333	89 (72)
Diabetes, n (%)	27 (38)	24 (46)	0.366	51 (42)
ACS, n (%)	61 (86)	41 (79)	0.303	102 (83)
Smoking history, n (%)	33 (47)	28 (54)	0.419	61 (50)
Previous stroke, n (%)	9 (13)	2 (4)	0.090	11 (9)
Previous AF, n (%)	3 (4)	4 (8)	0.412	7 (6)
*Blood test data*				
PO_2_ (mmHg)	94.2 ± 18.2	88.9 ± 25.5	0.183	92.0 ± 21.7
PCO_2_ (mmHg)	35.5 ± 3.2	37.1 ± 4.4	0.033*	36.2 ± 3.8
HDL (mmol/L)	1.1 ± 0.3	1.0 ± 0.3	0.273	1.0 ± 0.3
LDL (mmol/L)	2.4 ± 1.0	2.5 ± 1.1	0.677	2.4 ± 1.0
Triglyceride (mmol/L)	1.3 (1.1, 1.9)	1.3 (1.0, 1.8)	0.916	1.3 (1.1, 1.8)
Creatinine (µmol/l)	73.3 ± 17.8	76.8 ± 21.4	0.319	74.8 ± 19.4
Hs-CRP (mg/L)	1.1 (0.3, 2.2)	2.0 (0.7, 6.8)	0.001*	1.4 (0.5, 3.5)
*Echocardiography data*				
LVEF (%)	59.5 ± 8.5	55.9 ± 9.8	0.034*	58.0 ± 9.2
LVDD (mm)	48.9 ± 6.7	50.0 ± 5.9	0.326	49.4 ± 6.4
LAD (mm)	36.7 ± 3.8	37.0 ± 4.2	0.645	36.9 ± 4.0
*PG test data*				
AHI (events/h)	7.1 ± 4.1	28.5 ± 11.5	< 0.001*	16.1 ± 13.4
Mean apnea time (s)	17 (14, 24)	24 (19, 30)	< 0.001*	20 (16, 27)
Maximum apnea time (s)	27 (19, 35)	40 (31, 61)	< 0.001*	31 (22, 47)
Lowest SaO_2_ (%)	87.0 ± 3.7	81.6 ± 5.8	< 0.001*	84.7 ± 5.4
Mean SaO_2_ (%)	95.4 ± 2.1	93.0 ± 2.4	< 0.001*	94.4 ± 2.5
ODI ≥ 3%	6.1 (3.0, 9.3)	23.3 (18.4, 31.1)	< 0.001*	15.2 ± 13.5

Values are mean (± SD), median (interquartile range), or no. (%)

BMI: body mass index, ACS: acute coronary syndrome, AF: atrial fibrillation, PO_2_: partial pressure of oxygen, PCO_2_: partial pressure of carbon dioxide, HDL: high-density lipoprotein, LDL: low-density lipoprotein, Hs-CRP: high-sensitivity C-reactive protein, LVEF: left ventricular ejection fraction, LVDD: left ventricular end diastolic diameter, LAD: left atrium diameter, AHI: apnea hypopnea index, SaO_2_: arterial oxygen saturation, ODI: oxygen desaturation index

*P < 0.05

**Table 2:** Comparison of intraoperative and postoperative clinical data between patients with absent-mild obstructive sleep apnea (OSA) and those with moderate-severe OSA who underwent off-pump coronary artery bypass grafting (OPCABG)

	Absent-mild OSA (n = 71)	Moderate-severe OSA (n = 52)	*P* value	Total (n = 123)
No. of performed grafting	4.2 ± 0.6	4.2 ± 0.5	0.849	4.2 ± 0.5
Duration of surgery (min)	233.6 ± 35.7	229.4 ± 34.7	0.525	231.8 ± 35.2
MACCEs, n (%)	21 (30)	11 (21)	0.293	32 (26)
Lung infection, n (%)	2 (3)	2 (4)	0.751	4 (3)
POAF, n (%)	16 (23)	16 (31)	0.304	32 (26)
Duration of ventilator use (h)	21.1 ± 19.6	20.3 ± 13.3	0.799	20.8 ± 17.2
ICU stay (h)	34.0 ± 31.3	30.4 ± 22.8	0.478	32.5 ± 28.0
Duration of hospitalization (day)	17.2 ± 5.7	18.8 ± 6.7	0.176	17.9 ± 6.2
Hospital cost (× 1000 RMB)	128.4 ± 25.6	134.0 ± 28.7	0.260	130.8 ± 27.0

Values are mean (± SD), median (interquartile range), or no. (%)

MACCEs: major adverse cardiac or cerebrovascular events, POAF: postoperative atrial fibrillation

### Relationship between Hs-CRP level and severity of OSA

**Fig. 3:**
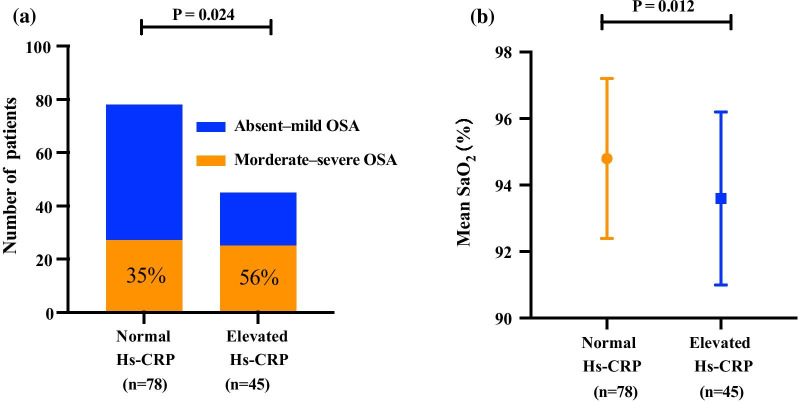
**a** The proportion of patients with moderate-severe OSA in the elevated Hs-CRP group was significantly higher than that in the normal Hs-CRP group. **b** The mean SaO_2_ in the elevated Hs-CRP group was significantly lower than that in the normal Hs-CRP group

A comparison of the severity of OSA between the normal Hs-CRP and elevated Hs-CRP groups (Fig. 3) showed that the proportion of patients with moderate-severe OSA was significantly higher in the elevated Hs-CRP group than in the normal Hs-CRP group (56% vs. 35%, *P* = 0.024), mean SaO_2_ in the elevated Hs-CRP group was significantly lower than that in the normal Hs-CRP group (93.6 ± 2.6 vs. 94.8 ± 2.4%, *P* = 0.012). Multivariate logistic regression showed that moderate-severe OSA was associated with elevated Hs-CRP level (dependent variable) (OR = 2.356, 95% CI 1.101–5.041, *P* = 0.027) after adjusting sex, age, and BMI.

### Correlation of Hs-CRP level with POAF, duration of hospitalization, and hospital cost

**Fig. 4:**
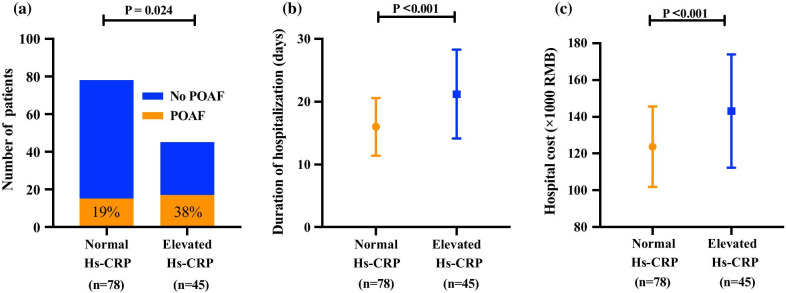
**a** The proportion of patients with POAF in the elevated Hs-CRP group was significantly higher than that in the normal Hs-CRP group. **b** The duration of hospitalization in the elevated Hs-CRP group was significantly longer than that in normal group. **c** The hospital cost in the elevated Hs-CRP group was significantly greater than that in the normal Hs-CRP group

A comparison of postoperative clinical data between the normal Hs-CRP and elevated Hs-CRP groups (Fig. 4) showed that the proportion of patients with POAF was significantly higher in the elevated Hs-CRP group than in the normal Hs-CRP group (38% vs. 19%, *P* = 0.024); duration of hospitalization (21.2 ± 7.1 vs. 16.0 ± 4.6 days, *P* < 0.001) and hospital cost (143.1 ± 30.7 vs. 123.7 ± 21.8 × 1000 RMB, *P* < 0.001) in the elevated Hs-CRP group were significantly higher than those in the normal Hs-CRP group.

Hs-CRP level was an independent risk factor for POAF (OR = 1.212, 95% CI 1.048–1.403, *P* = 0.01) (Table 3). Table 4 showed that hypertension (B = −2.522, 95% CI −4.833 to −0.211, *P* = 0.033), Hs-CRP level (B = 0.456, 95% CI 0.202–0.710, *P* = 0.001) and LVEF (B = −0.159, 95% CI −0.276 to −0.043, *P* = 0.008) independently correlated with the duration of hospitalization; hypertension (B = −12.851, 95% CI −22.677 to −3.025, *P* = 0.011), Hs-CRP level (B = 1.111, 95% CI 0.031–2.192, *P* = 0.044), and LVEF (B = −1.122, 95% CI −1.619 to −0.626, *P* < 0.001) independently correlated with hospital costs.

**Table 3:** Univariate and multivariate logistic regression analyses for the correlation between various preoperative clinical indicators and postoperative atrial fibrillation

Covariate	Univariate		Multivariate	
	Odds ratio (95% CI)	*P* value	Odds ratio (95% CI)	*P* value
Male	0.720 (0.288–1.800)	0.482	1.645 (0.465–5.821)	0.440
Age (years)	1.028 (0.980–1.078)	0.260	1.021 (0.964–1.081)	0.475
BMI (kg/m^2^)	1.043 (0.925–1.176)	0.491	1.109 (0.957–1.284)	0.168
Hypertension	1.507 (0.582–3.900)	0.398	2.044 (0.659–6.340)	0.216
Diabetes	0.668 (0.289–1.545)	0.346	0.375 (0.132–1.061)	0.064
Smoking history	0.430 (0.186–0.993)	0.048*	0.277 (0.096–0.804)	0.018*
Hs-CRP (mg/L)	1.166 (1.050–1.294)	0.004*	1.212 (1.048–1.403)	0.010*
LVEF (%)	0.949 (0.909–0.991)	0.017*	0.935 (0.881–0.994)	0.031*
LAD (mm)	1.095 (0.988–1.214)	0.083	1.051 (0.926–1.194)	0.440
AHI (events/h)	1.014 (0.985–1.044)	0.342	0.994 (0.956–1.032)	0.746

BMI: body mass index, PCO_2_: partial pressure of carbon dioxide, Hs-CRP: high-sensitivity C-reactive protein, LVEF: left ventricular ejection fraction, LAD: left atrium diameter, AHI: apnea hypopnea index

*P < 0.05

**Table 4:** Simple and multiple linear regression analysis for the correlation between various preoperative clinical indicators and postoperative atrial fibrillation, duration of hospitalization, and hospital cost

Covariate	Dependent variable: duration of hospitalization				Dependent variable: hospital cost			
	Univariate		Multivariate		Univariate		Multivariate	
	B (95% CI)	P value	B (95% CI)	P value	B (95% CI)	*P* value	B (95% CI)	*P* value
Male	0.868 (− 1.730 to 3.465)	0.510	1.867 (− 0.652 to 4.385)	0.145	− 0.327 (− 11.717 to 11.063)	0.955	3.999 (− 6.710 to 14.708)	0.461
Age (years)	− 0.026 (− 0.151 to 0.099)	0.678	− 0.015 (− 0.134 to 0.103)	0.796	0.270 (− 0.274 to 0.815)	0.328	0.346 (− 0.157 to 0.849)	0.176
BMI (kg/m^2^)	− 0.025 (− 0.348 to 0.299)	0.880	0.116 (− 0.181 to 0.413)	0.441	− 0.954 (− 2.360 to 0.452)	0.182	− 0.012 (− 1.276 to 1.252)	0.985
Hypertension	− 2.131 (− 4.570 to 0.309)	0.086	− 2.522 (− 4.833 to 0.211)	0.033*	− 13.934 (− 24.450 to 3.418)	0.010*	− 12.851 (− 22.677 to 3.025)	0.011*
Diabetes	0.457 (− 1.784 to 2.697)	0.687	− 0.154 (− 2.177 to 1.869)	0.880	1.819 (− 7.990 to 11.627)	0.714	− 0.894 (− 9.496 to 7.708)	0.837
Hs− CRP (mg/L)	0.501 (0.251 to 0.751)	< 0.001*	0.456 (0.202 to 0.710)	0.001*	1.080 (0.500 to 2.750)	0.005*	1.111 (0.031 to 2.192)	0.044*
LVEF (%)	− 0.235 (− 0.347 to 0.122)	< 0.001*	− 0.159 (− 0.276 to 0.043)	0.008*	− 1.346 (− 1.814 to 0.877)	< 0.001*	− 1.122 (− 1.619 to 0.626)	< 0.001*
AHI (events/h)	0.112 (0.031 to 0.192)	0.007*	0.068 (− 0.011 to 0.146)	0.090	0.441 (0.087 to 0.795)	0.015*	0.218 (− 0.116 to 0.552)	0.199

BMI: body mass index, PCO_2_: partial pressure of carbon dioxide, Hs-CRP: high-sensitivity C-reactive protein, LVEF: left ventricular ejection fraction, AHI: apnea hypopnea index

*P < 0.05

## Discussion

In this prospective cohort of 123 patients who underwent OPCABG, the prevalence of OSA was 42.3%. AHI, the criteria for severity of OSA, has limitations in predicting the prognosis of OPCABG. Hs-CRP level was closely related to the severity of OSA, and it independently correlated with POAF, duration of hospitalization and hospital cost.

OSA is closely related to CHD. A study showed that the prevalence of CHD in patients with OSA was 16.2% and that of patients without OSA was 5.4% [7]; in a population with suspected CHD, the proportion of patients with moderate-severe OSA was 24%, four times higher than the prevalence in the normal population [8]. In the present study, we found that the proportion of patients with moderate-severe OSA was significantly higher than that of the previous study. Patients undergoing OPCABG with severe coronary artery disease were included in this study, which potentially indicated that OSA was a substantial risk factor for CHD.

CABG is the standard of care for patients with extensive CHD. Few studies showed that OSA might affect the prognosis of patients undergoing CABG. Uchôa et al. found that OSA significantly increased the long-term incidence of MACCEs (follow-up time of 4.5 years), revascularization rate, the proportion of angina attacks, and AF incidence in patients with CABG; however, there was no significant effect on the 30-day prognosis after CABG [9]. Another study found that AHI was an independent risk factor for increased duration of hospitalization and postoperative circulatory fluctuation in patients with CABG [10]. In contrast, we found no significant difference in postoperative indicators between the moderate-severe OSA and absent-mild OSA groups. The possible reason for the discrepancy was that all patients included in our study underwent OPCABG, avoiding the effects of extracorporeal circulation, shortening the postoperative recovery cycle, which in turn reduced the short-term effect of OSA.

Evidence to identify the effect of OSA on the prognosis of OPCABG only using OSA classification is insufficient. OSA may affect the clinical outcome of CABG by influencing other indicators. For example, our previous study found that OSA might further affect the perioperative indicators such as the cardiac function [4]. In addition, biomarkers related to OSA and CHD may also be examined to predict more accurately the effect of OSA on CABG.

CRP is an acute phase reaction protein. Repeated hypoxia and inadequate ventilation in OSA could trigger oxidative stress and systemic inflammatory response, which could in turn enhance the synthesis and release of CRP [11]. Shamsuzzaman et al. showed that CRP has a significant linear correlation with AHI and is an independent influencing factor for OSA severity [12]. Moreover, inflammatory responses play key roles in the development of atherosclerosis. CRP, the product and mediator of inflammatory responses in atherosclerosis, is an important marker of endothelial dysfunction. Elevated CRP levels have been shown to be an independent risk factor for diseases such as myocardial infarction, peripheral vascular disease, and stroke [13]. Han et al. found that high CRP levels related to acute renal function injury, all-cause death, duration of hospitalization, and ICU stay after CABG [14].

Compared with the CRP, Hs-CRP extends the detection linear range from 3–200 to 0.005–0.10 mg/L, thereby making the determination of low-concentration CRP more accurate. Moreover, Hs-CRP has a long half-life, with no diurnal difference and no sex- or age-dependence and has a higher value in predicting the prognosis of cardiovascular and cerebrovascular diseases [15]. Previous studies have shown a relationship between Hs-CRP levels and OSA, nevertheless, these results are controversial because of obesity and various confounding factors [16]. In our study, we found that the Hs-CRP level was significantly increased in the moderate-severe OSA group, and there were significant correlations between Hs-CRP level and severity of OSA after adjusting sex, age, and BMI.

Hs-CRP plays an important role in predicting the prognosis of cardiovascular disease; nevertheless, only one report on the early effect of Hs-CRP on OPCABG has been conducted [17]. In the present study, we found that elevated Hs-CRP levels were significantly associated with increased AF incidence and duration of hospitalization. Our results also demonstrated that AF incidence, duration of hospitalization, and hospital costs were significantly higher in the elevated Hs-CRP group than in the normal group after OPCABG. Further regression analysis showed that Hs-CRP level was an independent risk factor for POAF and was independently correlated with the duration of hospitalization and hospital cost.

In addition, stability of respiratory regulation is an important factor in determining OSA severity [18]. Compared with the normal population, patients with OSA had significantly reduced respiratory center responses to low PO_2_ and high PCO_2_ during sleep, and the respiratory center response of some patients was also suppressed during wakefulness. Moreover, patients with OSA have long durations of apnea at night with short intervals. While hyperventilation occurs at the end of an apneic event, it is insufficient to clear the accumulated CO_2_, thereby resulting in hypercapnia or even type II respiratory failure. In this study, we found that the periods of apnea were significantly longer and the PCO_2_ level was significantly higher in the moderate-severe OSA group than in the absent-mild OSA group when awake. Further study involving the change in PCO_2_ and internal environment is needed.

In this sudy, the preoperative LVEF of patients with moderate-severe OSA was significantly lower than that of patients with absent-mild OSA. Previous studies also found that LVEF was independently related to moderate-severe OSA. The main mechanisms by which OSA affects cardiac function may be as follows [19, 20]: first, each respiratory obstruction event could result in an intrathoracic negative pressure of 60–70 cm H_2_O, and hypoxia could cause pulmonary vasoconstriction, resulting in preload and afterload imbalance between the left and right ventricles; subsequently, myocardial oxygen consumption increases and myocardial ischemia occurs, which in turn alters cardiac function; second, the long-term repeated fluctuation of intrathoracic pressure could increase intraglomerular pressure variability, leading to impaired cardiac function; and finally, sympathetic hyperactivity affects all-day cardiopulmonary hemodynamics. In addition, hypopharyngeal edema due to decreased cardiac function could also promote the development of OSA.

CPAP remains the standard of care for patients with OSA, and evidence has revealed that it is correlated with a reduced inflammatory response [21]. Studies have confirmed that CPAP might result in decreased risk of repeat revascularization and cardiac death of patients with OSA after percutaneous coronary intervention (PCI) [22, 23]. However, we know little about the role of CPAP in improving prognosis of CABG. In consideration of our previous clinical practice, patient compliance and timing of CPAP treatment after CABG might be the key to curative effects. Additional trials evaluating the effects of CPAP in patients with OSA who underwent CABG are warranted.

Our study has the following limitations: first, it is a single-center study with a limited sample size; the results need to be validated using multi-center, large-sample studies. Second, we were unable to use polysomnography (PSG), which appears to offer a more accurate evaluation of OSA than does PG. In addition, PG may underestimate the OSA severity. PSG requires patients to sleep at the sleep center for more than 8 h. The sleep center in our hospital has not been provided with urgent response equipment and medical teams for patients with heart disease. All of our patients had severe coronary artery disease and were consequently at a high risk of emergent cardiac events. Therefore, we decided to use a portable PG monitor so that we could keep our patients in the cardiac surgery department for the assessment of OSA. Finally, we only analyzed Hs-CRP levels at one time point; thus, it remains unclear as to whether Hs-CRP level has the same predictive value at other time points.

## Conclusions

The results of our study suggested that OSA, particularly moderate-severe OSA, was highly prevalent in patients undergoing OPCABG. Compared with patients with absent-mild OSA, those with moderate-severe OSA had poorer cardiac function and higher PCO_2_ and Hs-CRP levels. The Hs-CRP level was closely related to the severity of OSA and has an important predictive value for POAF, duration of hospitalization, and hospital cost among patients with OPCABG.

## Data Availability

The datasets used or analyzed during the current study are available from the corresponding author on reasonable request.

## Abbreviations

OSA: Obstructive sleep apnea
Hs-CRP: High-sensitivity C-reactive protein
OPCABG: Off-pump cardiac artery bypass grafting
PO_2_: Partial pressure of oxygen
PCO_2_: Partial pressure of carbon dioxide
AHI: Apnea hypopnea index
ODI: Oxygen desaturation index
LVEF: Left ventricle ejection fraction
SaO_2_: Arterial oxygen saturation
POAF: Post-CABG atrial fibrillation
CHD: Coronary heart disease
PG: Polygraphy
BMI: Body mass index
ICU: Intensive care unit
MACCE: Major adverse cardiac and cerebrovascular event
ACS: Acute coronary syndrome
HDL: High-density lipoprotein
LDL: Low-density lipoprotein
LVDD: Left ventricular end diastolic diameter
LAD: Left atrium diameter
